# Association of SARS-CoV-2 With Health-related Quality of Life 1 Year After Illness Using Latent Transition Analysis

**DOI:** 10.1093/ofid/ofaf278

**Published:** 2025-06-10

**Authors:** Lauren E Wisk, Michael Gottlieb, Peizheng Chen, Huihui Yu, Kelli N O’Laughlin, Kari A Stephens, Graham Nichol, Juan Carlos C Montoy, Robert M Rodriguez, Michelle Santangelo, Kristyn Gatling, Erica S Spatz, Arjun K Venkatesh, Kristin L Rising, Mandy J Hill, Ryan Huebinger, Ahamed H Idris, Michael Willis, Efrat Kean, Samuel A McDonald, Joann G Elmore, Robert A Weinstein

**Affiliations:** Division of General Internal Medicine and Health Services Research, David Geffen School of Medicine, University of California, Los Angeles, Los Angeles, California, USA; Department of Health Policy and Management, Fielding School of Public Health, University of California, Los Angeles, Los Angeles, California, USA; Department of Emergency Medicine, Rush University Medical Center, Chicago, Illinois, USA; Section of Cardiovascular Medicine, Center for Outcomes Research and Evaluation (CORE), Yale School of Medicine, New Haven, Connecticut, USA; Section of Cardiovascular Medicine, Center for Outcomes Research and Evaluation (CORE), Yale School of Medicine, New Haven, Connecticut, USA; Department of Emergency Medicine, University of Washington, Seattle, Washington, USA; Department of Global Health, University of Washington, Seattle, Washington, USA; Department of Family Medicine, University of Washington, Seattle, Washington, USA; Harborview Center for Prehospital Emergency Care, University of Washington, Seattle, Washington, USA; Department of Emergency Medicine, University of California, San Francisco, San Francisco, California, USA; Department of Medicine, University of California Riverside School of Medicine, Riverside, California, USA; Department of Emergency Medicine, Rush University Medical Center, Chicago, Illinois, USA; Department of Internal Medicine, Division of Infectious Diseases, Rush University Medical Center, Chicago, Illinois, USA; Section of Cardiovascular Medicine, Center for Outcomes Research and Evaluation (CORE), Yale School of Medicine, New Haven, Connecticut, USA; Section of Cardiovascular Medicine, Department of Medicine, Yale School of Medicine, New Haven, Connecticut, USA; Section of Cardiovascular Medicine, Center for Outcomes Research and Evaluation (CORE), Yale School of Medicine, New Haven, Connecticut, USA; Department of Emergency Medicine, Yale School of Medicine, New Haven, Connecticut, USA; Department of Emergency Medicine, Thomas Jefferson University, Philadelphia, Pennsylvania, USA; Center for Connected Care, Thomas Jefferson University, Philadelphia, Pennsylvania, USA; Department of Emergency Medicine, McGovern Medical School, UT Health Houston, Houston, Texas, USA; Department of Emergency Medicine, McGovern Medical School, UT Health Houston, Houston, Texas, USA; Department of Emergency Medicine, UT Southwestern Medical Center, Dallas, Texas, USA; Department of Neurology, University of Washington, Seattle, Washington, USA; Department of Emergency Medicine, Thomas Jefferson University, Philadelphia, Pennsylvania, USA; Center for Connected Care, Thomas Jefferson University, Philadelphia, Pennsylvania, USA; Department of Emergency Medicine, UT Southwestern Medical Center, Dallas, Texas, USA; Clinical Informatics Center, UT Southwestern Medical Center, Dallas, Texas, USA; Division of General Internal Medicine and Health Services Research, David Geffen School of Medicine, University of California, Los Angeles, Los Angeles, California, USA; Department of Internal Medicine, Division of Infectious Diseases, Rush University Medical Center, Chicago, Illinois, USA; Division of Infectious Diseases, Department of Medicine, Cook County Hospital, Chicago, Illinois, USA

**Keywords:** COVID-19, health-related quality of life, prospective cohort study, SARS-CoV-2

## Abstract

**Background:**

Long-term sequelae after SARS-CoV-2 infection may impact health-related quality-of-life (HRQoL), yet it is unknown how HRQoL changes during recovery. We compared patient-reported HRQoL among adults with COVID-19–like illness who tested SARS-CoV-2 positive (COVID+) with those who tested negative (COVID−).

**Methods:**

Participants in this prospective, multicenter, longitudinal registry study were enrolled from December 2020 through August 2022 and completed 3-month follow-up assessments until 12 months after enrollment. Participants were adults (≥18 years) with acute symptoms suggestive of COVID-19 who received a Food and Drug Administration–approved SARS-CoV-2 test. Participants received questions from PROMIS-29 (subscales: physical function, anxiety, depression, fatigue, social participation, sleep disturbance, and pain interference) and PROMIS SF-8a (cognitive function). Latent transition analysis was used to identify meaningful patterns in HRQoL scores over time; 4 HRQoL categories were compared descriptively and using multivariable regression. Inverse probability weighting was used to adjust for covariate imbalance.

**Results:**

There were 1096 (75%) COVID+ and 371 (25%) COVID−. Four distinct well-being classes emerged: optimal overall, poor mental, poor physical, and poor overall HRQoL. COVID+ participants were more likely to return to the optimal HRQoL class compared to COVID− participants. The most substantial transition from poor physical to optimal HRQoL occurred by 3 months, whereas movement from poor mental to optimal HRQoL occurred by 9 months.

**Conclusions:**

In adults with COVID-19–like illness, COVID+ participants demonstrated meaningful recovery in their physical HRQoL by 3 months after infection, but mental HRQoL took longer to improve. Suboptimal HRQoL at 3 to 12 months after infection remained in approximately 20%.

**Trial Registration:**

NCT04610515.

## Teaser

Among adults with symptomatic illness, both COVID+ and COVID− participants reported improvements in HRQoL but recovery trajectories differ for physical versus mental HRQoL, with slower mental HRQoL recovery after illness. Persistently poor HRQoL was observed for those self-reporting long COVID.

Post-COVID conditions (often referred to as long COVID) include a heterogenous group of conditions not attributable to another cause that are present at least 3 months after SARS-CoV-2 infection [1–3]. Prior work has demonstrated prominent features of persistent fatigue and cognitive deficits, though reported symptoms are myriad [4–7].

While understanding the incidence and magnitude of discrete symptoms of long COVID and their individual trajectory is important, it is critical to better understand the longer term effect on health-related quality of life (HRQoL). Prior work demonstrated that people with persistent symptoms after COVID-19 had poor physical, mental, or social function at 3-month follow-up [7]. Such sequelae can have a profound impact on HRQoL, return to work, and other activities [8]. Well-established tools for measuring the impact of symptoms on patients' HRQoL include the Patient-Reported Outcomes Measurement Information System (PROMIS) instruments, which were developed and validated to evaluate patient-centric health domains like pain, fatigue, physical functioning, sleep, and emotional distress that can have a major impact on HRQoL [9, 10]. Specifically, evaluating the trajectory of HRQoL can provide broader insights into the mechanisms of recovery after infections than analysis of individual symptoms and may reveal longer term decrements in HRQoL.

The Innovative Support for Patients with SARS-CoV-2 Infections Registry (INSPIRE) was designed to prospectively assess long-term outcomes of adults with acute COVID-19 alongside contemporary controls comprising adults who had similar symptoms but tested negative for SARS-CoV-2 [11]. In this analysis, we describe the longitudinal changes in patient-reported outcomes of physical and mental HRQoL among symptomatic participants who tested positive or negative for COVID-19.

## METHODS

### Study Design and Data Source

INSPIRE was a prospective, multicenter, longitudinal study that enrolled individuals with acute symptoms (at least 1 of the following: fever >100.4 °F [38 °C]; feeling hot or feverish; chills; repeated shaking with chills; more tired than usual; muscle aches; joint pains; runny nose; sore throat; a new cough, or worsening of a chronic cough; shortness of breath; wheezing; pain or tightness in your chest; palpitations; nausea or vomiting; headache; hair loss; abdominal pain; diarrhea [>3 loose/looser than normal stools/24 hours]; decreased smell or change in smell; decreased taste or change in taste) [11] suggestive of COVID-19 in 8 sites across the United States. Recruitment occurred in person, by phone or email and through online advertisement. Participants were enrolled from December 2020 through August 2022, with follow-up through March 2023. A secure online platform (Hugo, Hugo Health LLC, Guilford, CT) facilitated the collation of consent-related materials, linkage to participants' electronic health records, and responses to self-administered surveys. This study was funded by the Centers for Disease Control and Prevention (CDC). This study was reviewed and approved by the Rush University, Washington University, University of California Los Angeles, University of California San Francisco, University of Texas Houston, University of Texas Southwestern, Yale University, and Thomas Jefferson University institutional review board (see 45 C.F.R. part 46.101(c), 25 C.F.R part 56), and all participants provided informed consent to participate. A detailed description of the study design was published [11]. This report follows the Strengthening the Reporting of Observational Studies in Epidemiology guidelines for cohort studies [12].

### Cohort Definition

This study included adults fluent in English or Spanish, with self-reported symptoms suggestive of acute SARS-CoV-2 infection and who were tested for SARS-CoV-2 with any US Food and Drug Administration–approved or authorized molecular or antigen-based assay during the 42 days before enrollment.

For the present analysis, participants were grouped based on their initial COVID-19 test result as COVID+ or COVID−. If more than 1 SARS-CoV-2 test was performed within 7 days of enrollment and results were discordant, we considered the positive test to be the true result. However, if a person's test positivity changed during the study (ie, after 7 days after enrollment), we retained them in their initial group.

**Figure 1.**
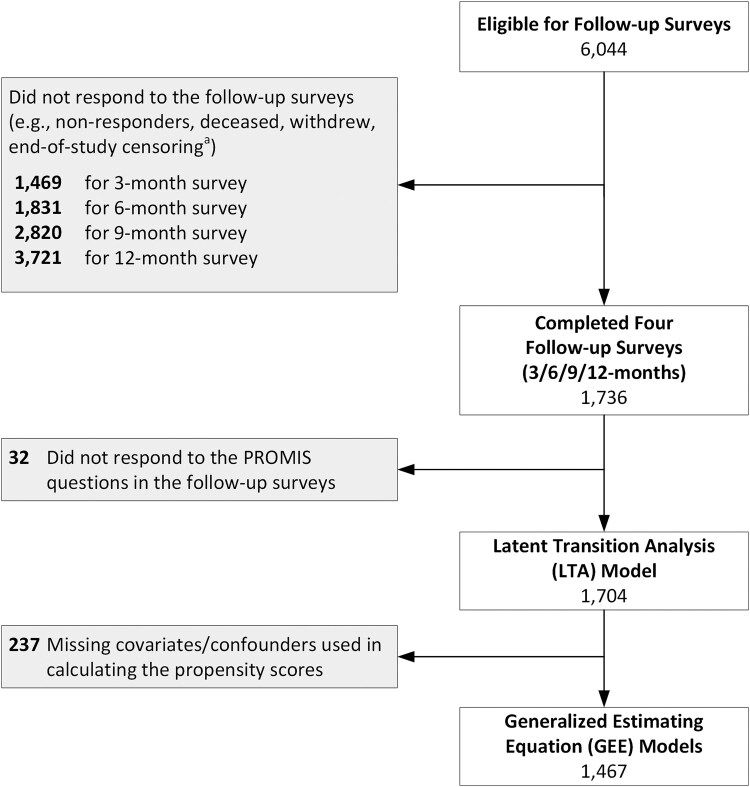
Participant flow diagram. ^a^The data collection ended on 28 February 2023. Specifically, the numbers of surveys censored by the end of study were: 0 for 3 months, 3 for 6 months, 1089 for 9 months, and 2269 for 12 months.

To support the latent transition analysis, we included all participants who completed all assessments during the first 12 months of follow-up (conducted every 3 months after the baseline assessment); see flow diagram (Figure 1). Participants included in this analysis were enrolled from 7 December 2020 to 29 August 2022. To evaluate for potential nonresponse bias that may have been introduced by including only those with complete data across the first 5 survey waves, we evaluated baseline characteristics among our analytic sample to the remainder of the registry enrolled sample (ie, those lost to follow-up at any point in the first 12 months; Supplementary Table 1). Participants lost to follow-up were more likely to be non-White, of lower educational attainment, never married, and receive their COVID-19 test using at at-home kit but were less likely to have several conditions (including asthma, diabetes, hypertension, overweight/obesity) or be a current tobacco user.

### Cohort Characteristics

Participants self-reported sociodemographic data at baseline, including age, gender, ethnicity, race, educational level, marital status, income, health insurance, and employment. Participants also provided information on chronic conditions, location of their SARS-CoV-2 test, and COVID-19–like symptoms. The latter were assessed using questions derived from the CDC Person Under Investigation for SARS-CoV-2 survey [13]. Self-reported race and ethnicity data were included because SARS-CoV-2 infection, testing, and outcomes varied across racial and ethnic groups [14].

### Outcomes

We defined outcomes as the classes identified using latent transition analysis (LTA); this method was selected as it allowed us to model the interrelations of individual PROMIS domains over time such that we could derive discrete classes of HRQoL that are consistently observed across follow-up. Latent classes were derived using LTA models incorporating the PROMIS T-scores of 8 domains from baseline to 12 months, with data points at 3-month intervals between a baseline survey and 4 follow-up surveys. Specifically, for 7 of the PROMIS-29 subscales (physical function, anxiety, depression, fatigue, social participation, sleep disturbance, and pain interference) and the PROMIS SF-CF 8a (cognitive function), we calculated the raw scores and used the crosswalk to get the PROMIS T-scores on a scale with a mean of 50 and a standard deviation of 10 among the US general population [9, 15]. We tested LTA models with 2 to 6 classes at each time point, examining their model fits (ie, which had the lowest Akaike Information Criteria and Bayesian Information Criteria and entropies greater than 0.8 across all time points).

### Statistical Analysis

We compared sociodemographic and clinical characteristics of the COVID-19 groups (COVID+ vs COVID−) using chi-squared tests or Fisher's exact tests, as appropriate, for categorical variables and *t*-tests for continuous variables (Table 1). To address any significant differences between the COVID-19 groups in baseline characteristics, we employed inverse propensity score weighting (IPW); covariates were selected for IPW models if they were bivariately related (*P* < .05) to both COVID-19 status (Supplementary Table 2) and latent classes (Supplementary Table 3). We examined the standardized mean difference in characteristics between COVID-19 groups to ensure balance of measured potential confounders through IPW (Supplementary Figure 1). To examine the difference in the probability of latent classes by COVID-19 status at each timepoint, we employed generalized estimating equations (GEE) models with IPW and adjusted for the status of new positive SARS-CoV-2 test results reported by each timepoint; across all time points, participants who were COVID– at baseline had higher rates of subsequent positive COVID tests (Supplementary Table 2). To characterize shifts among the latent classes over time, we conducted GEE modeling with IPW and adjusted for the latent classes at a prior time; from these models, we calculated the first-order transition probability of moving from a given class at time period t to the optimal HRQoL class at time period t + 1 to identify when recovery (ie, movement into the optimal class) was most likely to occur, by COVID-19 status.

**Table 1.** Sociodemographic and Clinical Characteristics of Symptomatic Adults Who Tested Positive (COVID+) vs Negative (COVID−) for SARS-CoV-2 at Enrollment, Weighted^a^

Characteristics^b^	Overall	COVID+	COVID−	*P* Value^c^
	N = 1467	N = 733	N = 734	
**Age (at enrollment)**	…	…	…	.882
18–34	608 (41.4%)	305 (41.6%)	302 (41.2%)	…
35–49	459 (31.3%)	228 (31.1%)	231 (31.5%)	…
50–64	266 (18.1%)	138 (18.9%)	128 (17.4%)	…
65+	134 (9.1%)	62 (8.4%)	72 (9.9%)	…
**Gender**	…	…	…	.428
Female	1033 (70.4%)	503 (68.5%)	531 (72.4%)	…
Male	414 (28.3%)	221 (30.1%)	194 (26.4%)	…
Transgender/nonbinary/other	19 (1.3%)	10 (1.4%)	9 (1.2%)	…
**Ethnicity**	…	…	…	.041
No, not of Hispanic, Latin, or Spanish origin	1231 (84.7%)	633 (87.3%)	598 (82.2%)	…
Yes, of Hispanic, Latin, or Spanish origin	222 (15.3%)	92 (12.7%)	129 (17.8%)	…
Unknown	14	7	7	…
**Race**	…	…	…	.906
White	1021 (71.5%)	507 (70.5%)	514 (72.5%)	…
Black or African American	116 (8.1%)	58 (8.1%)	58 (8.2%)	…
Asian	163 (11.4%)	86 (12.0%)	77 (10.9%)	…
Other/multiple	127 (8.9%)	68 (9.4%)	60 (8.4%)	…
Unknown	39	14	25	…
**Educational attainment**	…	…	…	.991
Less than high school diploma	11 (0.7%)	6 (0.8%)	5 (0.7%)	…
High school graduate or GED	100 (6.8%)	48 (6.6%)	52 (7.0%)	…
Some college but did not complete degree	202 (13.7%)	106 (14.4%)	96 (13.1%)	…
2-y college degree	123 (8.4%)	60 (8.1%)	63 (8.6%)	…
4-y college degree	499 (34.0%)	247 (33.7%)	252 (34.4%)	…
More than 4-y college degree	532 (36.3%)	266 (36.3%)	266 (36.2%)	…
**Marital status**	…	…	…	.969
Never married	461 (31.4%)	230 (31.3%)	231 (31.5%)	…
Married/living with a partner	839 (57.2%)	422 (57.5%)	417 (56.9%)	…
Divorced/widowed/separated	167 (11.4%)	82 (11.1%)	85 (11.6%)	…
**Family income (prepandemic)**	…	…	…	.882
Less than $10 000	94 (6.4%)	47 (6.4%)	47 (6.4%)	…
$10 000–$35 000	178 (12.2%)	90 (12.2%)	89 (12.1%)	…
$35 000 to less than $50 000	147 (10.0%)	79 (10.8%)	68 (9.3%)	…
$50 000 to less than $75 000	234 (15.9%)	109 (14.8%)	125 (17.1%)	…
$75 000 or more	814 (55.5%)	409 (55.8%)	405 (55.2%)	…
**Where received COVID test**	…	…	…	.938
At-home testing kit	85 (5.8%)	43 (5.9%)	42 (5.7%)	…
Tent/drive-up testing site	810 (55.2%)	410 (55.9%)	400 (54.5%)	…
Clinic including an urgent care clinic	270 (18.4%)	134 (18.3%)	136 (18.5%)	…
Hospital	113 (7.7%)	59 (8.0%)	54 (7.3%)	…
Emergency department	81 (5.5%)	35 (4.8%)	46 (6.3%)	…
Other	108 (7.3%)	52 (7.1%)	56 (7.6%)	…
**Health insurance**	…	…	…	.884
Private and public	61 (4.2%)	28 (3.9%)	33 (4.4%)	…
Private only	1055 (71.9%)	531 (72.5%)	524 (71.3%)	…
Public only	309 (21.1%)	150 (20.5%)	159 (21.7%)	…
None	42 (2.8%)	23 (3.2%)	18 (2.5%)	…
**Including yourself, how many adults over the age of 65 years are living in your household?**	…	…	…	.871
None	1212 (82.7%)	611 (83.3%)	601 (82.0%)	…
One	156 (10.7%)	74 (10.1%)	82 (11.2%)	…
More than 1	98 (6.7%)	48 (6.6%)	50 (6.8%)	…
**Employed before the pandemic**	…	…	…	.323
No	289 (19.7%)	134 (18.3%)	155 (21.1%)	…
Yes	1178 (80.3%)	599 (81.7%)	579 (78.9%)	…
**Was a non-health essential worker**	…	…	…	.888
No	840 (71.3%)	429 (71.6%)	411 (71.0%)	…
Yes	338 (28.7%)	170 (28.4%)	168 (29.0%)	…
Unknown	289	134	155	…
**Baseline (acute illness): sickness severity (0–10)**	5.71 (2.60)	5.68 (2.50)	5.73 (2.71)	.485
**Asthma (moderate or severe)**	…	…	…	.881
No	1259 (85.8%)	631 (86.0%)	629 (85.7%)	…
Yes	208 (14.2%)	102 (14.0%)	105 (14.3%)	…
**Heart conditions, such as coronary artery disease, heart failure, or cardiomyopathies**	…	…	…	.680
No	1433 (97.7%)	715 (97.5%)	718 (97.9%)	…
Yes	34 (2.3%)	18 (2.5%)	16 (2.1%)	…
**Diabetes**	…	…	…	.729
No	1388 (94.6%)	696 (94.9%)	692 (94.3%)	…
Yes	79 (5.4%)	37 (5.1%)	42 (5.7%)	…
**Hypertension or high blood pressure**	…	…	…	.537
No	1277 (87.0%)	633 (86.4%)	644 (87.7%)	…
Yes	190 (13.0%)	100 (13.6%)	90 (12.3%)	…
**Overweight or obesity**	…	…	…	.665
No	1043 (71.1%)	526 (71.8%)	517 (70.4%)	…
Yes	424 (28.9%)	207 (28.2%)	217 (29.6%)	…
**Smoking (currently smoking any type of tobacco, including smokeless tobacco)**	…	…	…	.485
No	1394 (95.0%)	694 (94.6%)	701 (95.5%)	…
Yes	73 (5.0%)	40 (5.4%)	33 (4.5%)	…

^a^Weighted by inverse propensity scores.

^b^We provided n (column %) for categorical variables and mean (SD) for baseline (acute illness) sickness severity (the only continuous variable ranging from 0 to 10).

^c^Chi-squared test with Rao and Scott's second-order correction; Wilcoxon rank-sum test for complex survey samples.

The LTA models were developed using Mplus version 8.9 [16] and the GEE models were developed using SAS version 9.4 (SAS, Inc., Cary NC). R version 4.3.3 (R Foundation, Vienna, Austria) and MS Excel (Microsoft, Redmond, WA) were adopted to generate tables and figures regarding participants' characteristics, latent outcomes, and model estimates. All tests were 2-sided with an alpha criterion of 0.05.

## RESULTS

The study sample included 1096 (75%) COVID+ and 371 (25%) COVID− participants (Supplementary Table 2). Participants were predominantly female (68.5%), non-Hispanic (87.3%), White (70.5%), married or partnered (57.5%), and privately insured (72.5%). After the application of IPW, COVID+ participants were similar to COVID− participants except with respect to ethnicity (COVID+ were less likely to be Hispanic; Table 1).

**Figure 2.**
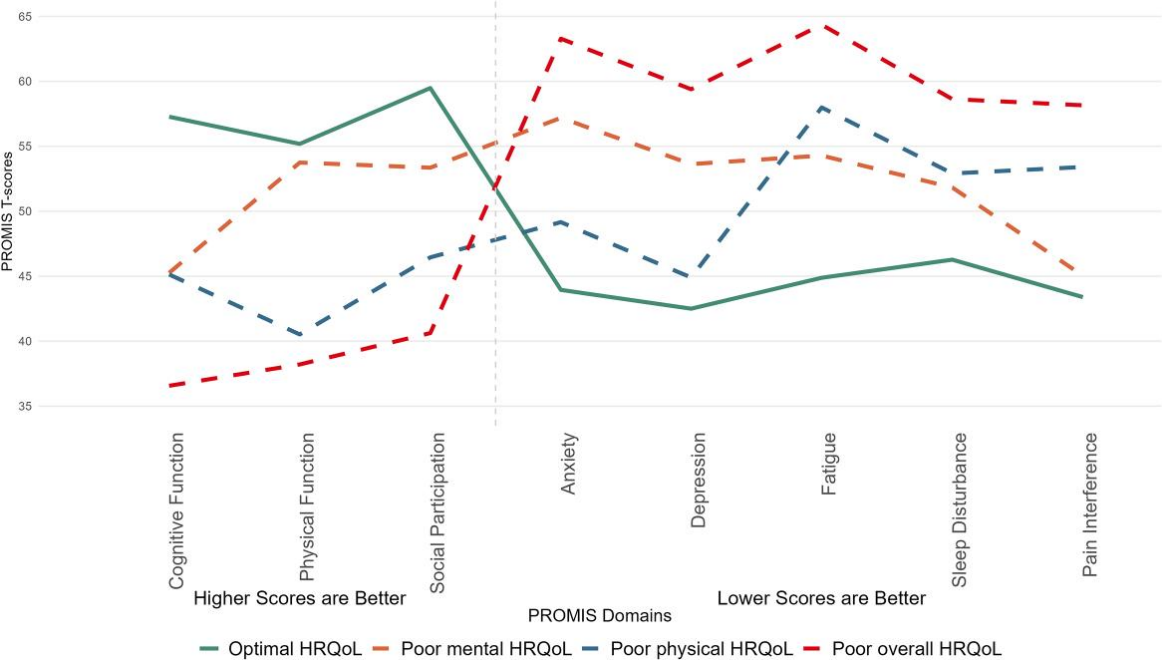
PROMIS domain scores at baseline, by latent class membership, among all participants. As latent transition analysis (LTA) was performed regardless of COVID status (ie, among all participants), results are shown inclusive of both the COVID+ and COVID− groups. Figure shows unweighted and unadjusted mean PROMIS scores (at baseline) on each domain included in the LTA, stratified by final class membership.

The best-fit LTA revealed 4 classes that were consistently identified across all timepoints (baseline through 12-month follow-up; Figure 2, Supplementary Figure 2). Based on the PROMIS domain scores, we named the 4 latent classes as: Optimal HRQoL, Poor mental HRQoL, Poor physical HRQoL, and Poor overall HRQoL. The first class comprised optimal HRQoL scores, which had the “best” mean (± standard deviation) scores across all domains: 57.3 ± 7.1 for cognitive function, 55.2 ± 4.4 for physical function, 59.5 ± 8.0 for social participation, 43.9 ± 5.5 for anxiety, 42.5 ± 3.5 for depression, 44.9 ± 7.1 for fatigue, 46.2 ± 6.3 for sleep disturbance, and 43.4 ± 4.6 for pain interference (all baseline). Poor mental HRQoL was characterized by poor scores for anxiety, depression, cognitive function, and fatigue (57.3 ± 5.9, 53.6 ± 6.1, 45.2 ± 7.8, and 54.3 ± 5.5 at baseline, respectively). Poor physical HRQoL was characterized by poor scores for cognitive function, physical function, social participation, fatigue, sleep disturbance, and pain interferences (45.1 ± 9.2, 40.4 ± 6.4, 46.5 ± 9.5, 58.0 ± 6.0, 53.0 ± 6.6, and 53.5 ± 8.6 at baseline, respectively). Poor overall HRQoL had the “worst” mean scores across all domains: 36.6 ± 7.1 for cognitive function, 38.2 ± 7.3 for physical function, 40.6 ± 8.4 for social participation, 63.3 ± 6.5 for anxiety, 59.4 ± 6.6 for depression, 64.4 ± 6.2 for fatigue, 58.8 ± 7.1 for sleep disturbance, and 58.2 ± 9.3 for pain interference (all baseline).

**Figure 3.**
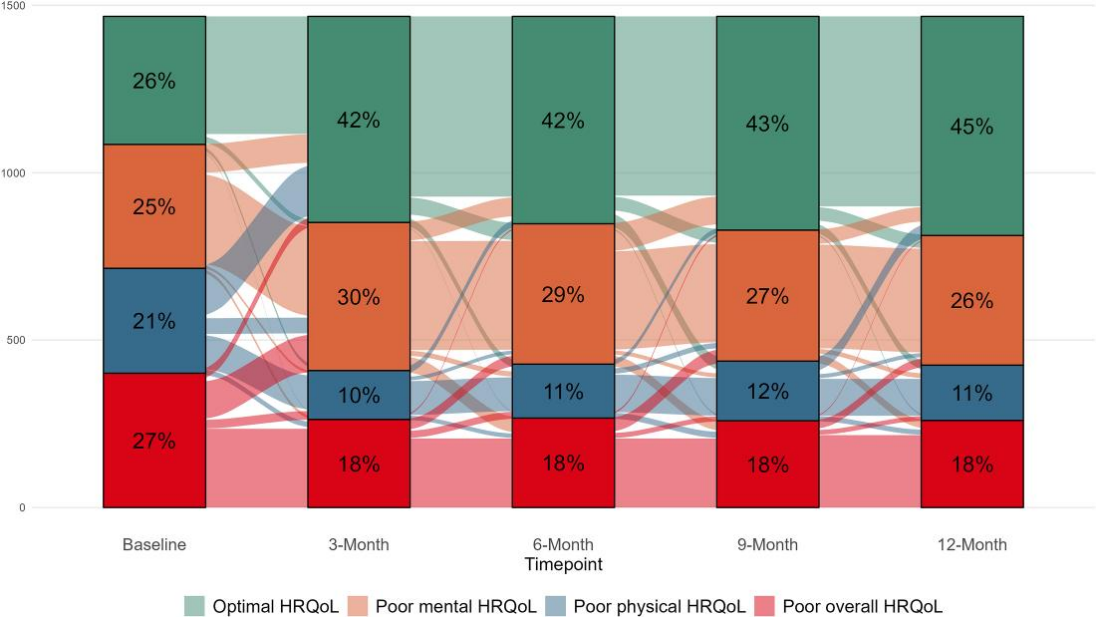
Sankey plot of latent transition analysis (LTA) membership across follow-up, among all participants. As LTA was performed irrespective of COVID status (ie, among all participants), results are shown inclusive of both the COVID+ and COVID− groups. Figure shows unweighted and unadjusted prevalence of final class membership at each time point from baseline to 12 months of follow-up.

At baseline, participants (including COVID+ and COVID−) were balanced across the 4 classes, with 73% of participants falling into 1 of the 3 classes with poor HRQoL (Figure 3). Class shifting frequently occurred between baseline and 3-month follow-up, with 58% of participants falling into a class with poor HRQoL at 3 months. Class shifting was less frequent at subsequent time points, and the overall portion of participants in each class remained largely consistent between 3 and 12 months. A substantial number of participants remained in the poor overall HRQoL class through 12 months after illness. Identified latent classes were substantially different with respect to several sociodemographic and clinical characteristics (Supplementary Table 3). Of note, participants in the poor physical HRQoL and poor overall HRQoL classes had significantly higher symptom severity scores for their acute illness at baseline, higher prevalence of comorbid conditions, and more symptoms at baseline.

Among those in the poor overall HRQoL class, 42.4% reported that they had long COVID at the final follow-up survey compared to 24.2% among those in the poor physical HRQoL class, 17.8% in the poor mental HRQoL class, and 9.7% in the optimal HRQoL class (*P* < .001; Supplementary Table 3).

**Figure 4.**
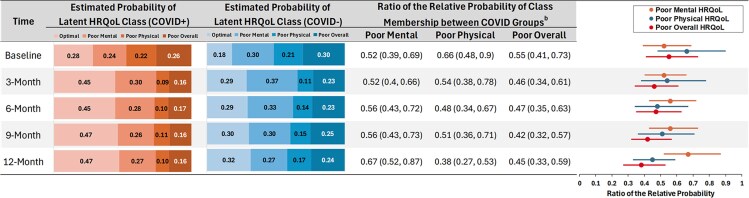
Forest plot demonstrating estimated^a^ associations between COVID-19 status with latent transition analysis membership at each time point. ^a^The estimated probabilities and ratios were derived from a GEE model accounting for the status of new positive SARS-CoV-2 test results reported by each timepoint (ie, subsequent COVID+ testing that occurred after baseline). Estimates also applied inverse probability weighting to adjust for differences between the COVID+ and COVID− groups. ^b^The ratio of the relative probability of poor HRQoL versus optimal HRQoL classes between COVID groups at each time point. Taking poor mental HRQoL as an example, ${\left(\frac{{\left(P_{\;poor\;mental}/P_{optimal}\right)}_{COVID+}}{{\left(P_{\;poor\;mental}/P_{optimal}\right)}_{COVID-}}\right)}_{t}$, where *t* denotes the 5 time points. HRQoL, health-related quality of life.

In adjusted models, the probability of being in the optimal HRQoL class increased over time in participants with and without COVID-19. Compared to the COVID− group, those in the COVID+ group had higher probability of being in the optimal HRQoL class and lower probability of being in the poor overall HRQoL class at baseline and all follow-up time points (Figure 4). In both COVID+ and COVID− groups, the probability of poor physical HRQoL decreased from baseline to 3 months and then remained relatively stable through 12 months. The probability of poor mental HRQoL, however, increased from baseline to 3 months and then gradually decreased from 3 through 12 months. Analysis of first-order transition probabilities (Supplementary Table 4) identified that recovery from baseline to 3 months (ie, moving to the optimal HRQoL class) was driven predominantly by improvements in physical HRQoL (eg, 50.8% of COVID+ individuals in the poor physical HRQoL class moved into the optimal HRQoL class between baseline and 3-month follow-up), whereas improvements in mental HRQoL were more pronounced between 6 and 9 months (eg, 20.6% of COVID+ individuals in the poor mental HRQoL class moved into the optimal HRQoL class between 6- and 9-month follow-up). The probability of recovering from poor overall well-being to optimal well-being was similar between COVID groups and generally low across time ranging between 1.4% and 5.2%.

## DISCUSSION

In this large, geographically diverse study of individuals with 12 months of follow-up after COVID-19-like illness, a substantial proportion of participants continued to report poor HRQoL, whether or not the inciting acute symptoms were due to SARS-CoV-2 or another illness. The majority of the recovery in physical HRQoL was observed within 3 months after acute illness, whereas recovery in mental well-being appeared to be more gradual, with significant improvements manifesting more profoundly between 6 and 9 months after infection. Importantly for patient prognostics, we found somewhat more pronounced recovery (ie, return to the optimal HRQoL) for those in the COVID+ group compared to the COVID− groups, after adjustment. Regardless, approximately 1 in 5 respondents remained in a poor overall HRQoL class with a high likelihood of self-reporting long COVID up to 12 months after initial infection.

As in prior work [7, 17, 18], we found similar reports of poor HRQoL after an acute illness for both individuals with a confirmed case of COVID-19 compared to those who tested COVID−, who also had poor baseline HRQoL. Poor HRQoL in the COVID– group may be attributable to heterogeneous conditions (or perhaps nonviral conditions related to work stress or general pandemic distress) and may also include those who had a false-negative COVID test or a subsequent SARS-CoV-2 infection [6] (which we previously demonstrated was more likely among our COVID− participants than our COVID+ participants). Thus, this comparison should be interpreted with caution given that the referent group (COVID−) likely includes individuals suffering from an unspecified illness(es) with varied expected recovery trajectories. We hypothesize that this biases our estimates toward the null. Still, several factors may explain the observed similarity between groups [19, 20]. First, prior medical research may have previously underestimated the prevalence of serious negative sequelae after acute illness, explaining why COVID+ and COVID− participants appear more similar after acute infection. Second, the study may be subject to selection bias, whereby only those COVID− participants presenting with more serious symptoms were enrolled and completed follow-up, leading to an overestimation of the HRQoL decrements seen in that cohort. Third, although we performed IPW to adjust for baseline differences between the cohorts that may confound findings, we did not have access to comprehensive medical histories that may also confound our comparison. For instance, preexisting conditions such as somatization may play a role in the observed well-being trajectories [21].

Recovery patterns based on different HRQoL domains (ie, physical vs mental) differed in important ways. Specifically, physical HRQoL (inclusive of poor scores for cognitive function, physical function, social participation, fatigue, sleep disturbance, and pain interferences) tended to show significant recovery as early as 3 months after infection, whereas mental HRQoL (inclusive of poor scores for anxiety, depression, cognitive function, and fatigue) exhibited a longer recovery window, with most of recovery to optimal HRQoL occurring between 6 and 9 months after acute illness. This aligns with prior reports that identify substantial change in the prevalence of discrete symptoms by 3 months after infection but stabilization thereafter [4, 6]. We are unaware of reports that outline mental HRQoL recovery after acute illness but our finding that these HRQoL domains have different recovery timelines supports the notion that treatment of mental health sequelae warrants special attention and may require longer periods of follow-up than previously identified using only reports of discrete symptoms to address outstanding patient concerns. Moreover, integrating routine HRQoL screening into post-COVID treatment encounters may be necessary to appropriately triage and provide mental health resources to individuals with lingering decrements in mental HRQoL after COVID.

Importantly, we identified a high prevalence and persistence of poor HRQoL up to 12 months in both the COVID+ and COVID− groups, suggesting a high societal burden. Across all patients, nearly one-fifth had poor overall HRQoL that remained consistent from 3 to 12 months. Nearly half of those in the poor overall HRQoL class self-reported long COVID thus indicating considerable potential overlap between those reporting impaired function and those with self-identified long COVID. Although it is challenging to separate the effects of the pandemic itself on these outcomes, the role of serious acute infections in inciting poor HRQoL warrants increased attention from the medical and public health sectors.

This analysis has several strengths, including multicenter recruitment of participants from diverse community, ambulatory, emergency, and inpatient settings; use of concurrent controls via recruiting symptomatic adults who tested negative for COVID-19; and prospective data collection using validated scales. Several limitations should also be noted. First, although this study aimed to recruit a diverse population across the United States, the requirement for access to a verifiable COVID-19 test, existing electronic health record system, and internet-enabled devices to administer study components may limit the generalizability of our sample. Furthermore, those with the most severe disease may have been unable/unwilling to participate or may have differentially dropped out across our mandatory 12-month follow-up window. Although we evaluated differences between those included versus excluded in our analytic sample, we may not have captured the full array of differences that would have contributed to an analytic sample with different class membership patterns over time. Second, it is unclear what heterogeneous acute condition symptomatic COVID− participants may have suffered from at the time of enrollment, making it difficult to hypothesize whether COVID− participants would be expected to have more or less severe patient-reported outcomes across time. Third, COVID-19 tests may yield false-negative or false-positive results [22, 23]; therefore, we cannot exclude the possibility that participants may have been misclassified (as either COVID+ or COVID−) based on their documented test result and that any such misclassification could attenuate differences in well-being observed between the 2 groups. Finally, the IPW approach was able to achieve balance between groups in measured covariates but we acknowledge that there may be imbalance among unmeasured factors that could have introduced residual confounding in our results.

## CONCLUSION

We identified important patterns in HRQoL recovery up to 1 year after infection with SARS-CoV-2, including a different recovery timeline for physical (faster recovery) versus mental HRQoL (slower recovery); underscoring the importance of providing mental health resources to individuals experiencing prolonged declines in mental HRQoL after COVID. SARS-CoV-2 infection was not associated with increasingly worse PROMIS scores across time and, in fact, COVID+ participants reported greater movement toward the optimal HRQoL class than did COVID− participants over a 12-month follow-up. Moreover, a high proportion of participants reported poor HRQoL up to 1 year after illness, suggesting that current treatment models may not be adequate to address lingering symptoms and their effects on quality of life, or that there is a disconnect between reported symptoms and quality of life that warrants a more nuanced approach to addressing recovery among those with COVID-19.

## Supplementary Material

ofaf278_Supplementary_Data

## Supplementary Data

Supplementary materials are available at *Open Forum Infectious Diseases* online. Consisting of data provided by the authors to benefit the reader, the posted materials are not copyedited and are the sole responsibility of the authors, so questions or comments should be addressed to the corresponding author.

## Notes

***Acknowledgments*.** The study sponsor was not involved in the conduct of the study or collection, management, or analysis of data. H.Y. and the Yale Analytic Core had full access to all the data in the study and were responsible for the integrity of the data and the accuracy of the data analysis. Partners from the Centers for Disease Control and Prevention (CDC; (Sharon Saydah, MHS, PhD, and Ian D. Plumb, MBBS, MSc) assisted with study design and the preparation of this manuscript. We also wish to acknowledge Kate Diaz Roldan, MPH, for her assistance with the references. See **Supplementary Acknowledgments** for a list of INSPIRE group members.

***Author Contributions*.** All author contributed to the conceptualization of the study. L.E.W., M.G., P.C., and H.Y. contributed to the methodologic approach and formal analysis. All authors contributed to the investigation, and subsequent review and editing. L.E.W. and M.G. take primary responsibility for the manuscript as a whole.

***Financial support.*** This work was supported by the Centers for Disease Control and Prevention (CDC) and the National Center of Immunization and Respiratory Diseases [contract number: 75D30120C08008 to R.A.W.]. Data are available upon reasonable request from the CDC.
