# Supplementary material for: Association of SARS-CoV-2 With Health-related Quality of Life 1 Year After Illness Using Latent Transition Analysis
Source: Open Forum Infect Dis. 2025 Jun 10;12(6):ofaf278. doi: 10.1093/ofid/ofaf278 (PMC12150399; doi:10.1093/ofid/ofaf278)
Supplement: ofaf278_Supplementary_Data [file ofaf278_supplementary_data.zip › Online-only material_2025.03.04_CLEAN_UPDATED.docx]

**Supplemental Online Content**

**Table of Contents**

**Supplementary Table 1.** Sociodemographic and Clinical Characteristics Comparing the Analytic Sample to the Registry Enrolled Sample, Unweighted

**Supplementary Table 2.** Sociodemographic and Clinical Characteristics of Symptomatic Adults Who Tested Positive (COVID +) vs Negative (COVID -) for SARS-CoV-2 at Enrollment, Unweighted

**Supplementary Table 3.** Sociodemographic and Clinical Characteristics of Symptomatic Adults by Latent Transition Analysis Membership, Unweighted

**Supplementary Figure 1.** Covariate Balance before and after Application of Inverse Probability Weighting

**Supplementary Figure 2.** PROMIS Domain Scores at Baseline, by Latent Class Membership, among All Participants

**Supplementary Table 4.** The estimated first order transition probability for latent classes at each time point, to the optimal HRQoL class in the next time point, by COVID-19 status

**Supplementary Acknowledgements**

**Supplementary Table 1.** Sociodemographic and Clinical Characteristics Comparing the Analytic Sample to the Registry Enrolled Sample, Unweighted

| Characteristics^a^ | Total | Included | Excluded | P-value^b^ |
| --- | --- | --- | --- | --- |
|  | (N=6044) | (N=1467) | (N=4577) |  |
| **Age (at enrollment)** |  |  |  |  |
| 18 to 34 | 2534 (41.9%) | 613 (41.8%) | 1921 (42.0%) | 0.992 |
| 35 to 49 | 1867 (30.9%) | 451 (30.7%) | 1416 (30.9%) |  |
| 50 to 64 | 1108 (18.3%) | 282 (19.2%) | 826 (18.0%) |  |
| 65+ | 491 (8.1%) | 121 (8.2%) | 370 (8.1%) |  |
| Missing | 44 (0.7%) | 0 (0%) | 44 (1.0%) |  |
| **Gender** |  |  |  |  |
| Female | 3972 (65.7%) | 1000 (68.2%) | 2972 (64.9%) | 0.968 |
| Male | 1818 (30.1%) | 446 (30.4%) | 1372 (30.0%) |  |
| Transgender/Non-binary/Other | 93 (1.5%) | 21 (1.4%) | 72 (1.6%) |  |
| Missing | 161 (2.7%) | 0 (0%) | 161 (3.5%) |  |
| **Ethnicity** |  |  |  |  |
| No, not of Hispanic, Latin or Spanish origin | 5026 (83.2%) | 1254 (85.5%) | 3772 (82.4%) | 0.173 |
| Yes, of Hispanic, Latin or Spanish origin | 903 (14.9%) | 199 (13.6%) | 704 (15.4%) |  |
| Missing | 115 (1.9%) | 14 (1.0%) | 101 (2.2%) |  |
| **Race** |  |  |  |  |
| White | 3922 (64.9%) | 1022 (69.7%) | 2900 (63.4%) | 0.001 |
| Black or African American | 616 (10.2%) | 110 (7.5%) | 506 (11.1%) |  |
| Asian | 767 (12.7%) | 171 (11.7%) | 596 (13.0%) |  |
| Other/Multiple | 543 (9.0%) | 134 (9.1%) | 409 (8.9%) |  |
| Missing | 196 (3.2%) | 30 (2.0%) | 166 (3.6%) |  |
| **Educational Attainment** |  |  |  |  |
| Less than high school diploma | 92 (1.5%) | 11 (0.7%) | 81 (1.8%) | 0.001 |
| High school graduate or GED | 536 (8.9%) | 93 (6.3%) | 443 (9.7%) |  |
| Some college but did not complete degree | 882 (14.6%) | 216 (14.7%) | 666 (14.6%) |  |
| 2-year college degree | 439 (7.3%) | 123 (8.4%) | 316 (6.9%) |  |
| 4-year college degree | 1848 (30.6%) | 490 (33.4%) | 1358 (29.7%) |  |
| More than 4-year college degree | 2110 (34.9%) | 534 (36.4%) | 1576 (34.4%) |  |
| Missing | 137 (2.3%) | 0 (0%) | 137 (3.0%) |  |
| **Marital Status** |  |  |  |  |
| Never married | 2255 (37.3%) | 466 (31.8%) | 1789 (39.1%) | <0.001 |
| Married/Living with a part | 3125 (51.7%) | 840 (57.3%) | 2285 (49.9%) |  |
| Divorced/Widowed/Separated | 658 (10.9%) | 161 (11.0%) | 497 (10.9%) |  |
| Missing | 6 (0.1%) | 0 (0%) | 6 (0.1%) |  |
| **Family Income (pre-pandemic)** |  |  |  |  |
| Less than $10,000 | 415 (6.9%) | 93 (6.3%) | 322 (7.0%) | 0.782 |
| $10,000 to $35,000 | 697 (11.5%) | 184 (12.5%) | 513 (11.2%) |  |
| $35,000 to less than $50,000 | 629 (10.4%) | 154 (10.5%) | 475 (10.4%) |  |
| $50,000 to less than $75,000 | 794 (13.1%) | 214 (14.6%) | 580 (12.7%) |  |
| $75,000 or more | 3073 (50.8%) | 822 (56.0%) | 2251 (49.2%) |  |
| Missing | 436 (7.2%) | 0 (0%) | 436 (9.5%) |  |
| **Where received COVID test** |  |  |  |  |
| At home testing kit | 812 (13.4%) | 84 (5.7%) | 728 (15.9%) | <0.001 |
| Tent/drive-up testing site | 2935 (48.6%) | 824 (56.2%) | 2111 (46.1%) |  |
| Clinic including an Urgent Care Clinic | 857 (14.2%) | 258 (17.6%) | 599 (13.1%) |  |
| Hospital | 553 (9.1%) | 122 (8.3%) | 431 (9.4%) |  |
| Emergency department | 361 (6.0%) | 70 (4.8%) | 291 (6.4%) |  |
| Other | 512 (8.5%) | 109 (7.4%) | 403 (8.8%) |  |
| Missing | 14 (0.2%) | 0 (0%) | 14 (0.3%) |  |
| **Health Insurance** |  |  |  |  |
| Private & Public | 196 (3.2%) | 57 (3.9%) | 139 (3.0%) | 0.324 |
| Private only | 4319 (71.5%) | 1063 (72.5%) | 3256 (71.1%) |  |
| Public only | 1282 (21.2%) | 300 (20.4%) | 982 (21.5%) |  |
| None | 247 (4.1%) | 47 (3.2%) | 200 (4.4%) |  |
| **Including yourself, how many adults over the age of 65 are living in your household?** |  |  |  |  |
| No | 4959 (82.0%) | 1223 (83.4%) | 3736 (81.6%) | 0.75 |
| One | 671 (11.1%) | 151 (10.3%) | 520 (11.4%) |  |
| More than 1 | 405 (6.7%) | 93 (6.3%) | 312 (6.8%) |  |
| Missing | 9 (0.1%) | 0 (0%) | 9 (0.2%) |  |
| **Employed before the pandemic** |  |  |  |  |
| No | 1197 (19.8%) | 263 (17.9%) | 934 (20.4%) | 0.113 |
| Yes | 4843 (80.1%) | 1204 (82.1%) | 3639 (79.5%) |  |
| Missing | 4 (0.1%) | 0 (0%) | 4 (0.1%) |  |
| **Was a non-health essential worker** |  |  |  |  |
| No | 3553 (58.8%) | 864 (58.9%) | 2689 (58.8%) | 0.342 |
| Yes | 1289 (21.3%) | 340 (23.2%) | 949 (20.7%) |  |
| Missing | 1202 (19.9%) | 263 (17.9%) | 939 (20.5%) |  |
| **Baseline (acute illness): sickness severity (0-10)** |  |  |  |  |
| Mean (SD) | 5.75 (2.61) | 5.63 (2.61) | 5.78 (2.61) | 0.153 |
| Missing | 9 (0.1%) | 0 (0%) | 9 (0.2%) |  |
| **Asthma (moderate or severe)** |  |  |  |  |
| No | 5385 (89.1%) | 1267 (86.4%) | 4118 (90.0%) | <0.001 |
| Yes | 566 (9.4%) | 200 (13.6%) | 366 (8.0%) |  |
| Missing | 93 (1.5%) | 0 (0%) | 93 (2.0%) |  |
| **Heart conditions, such as coronary artery disease, heart failure or cardiomyopathies** |  |  |  |  |
| No | 5833 (96.5%) | 1427 (97.3%) | 4406 (96.3%) | 0.063 |
| Yes | 118 (2.0%) | 40 (2.7%) | 78 (1.7%) |  |
| Missing | 93 (1.5%) | 0 (0%) | 93 (2.0%) |  |
| **Diabetes** |  |  |  |  |
| No | 5722 (94.7%) | 1389 (94.7%) | 4333 (94.7%) | 0.003 |
| Yes | 229 (3.8%) | 78 (5.3%) | 151 (3.3%) |  |
| Missing | 93 (1.5%) | 0 (0%) | 93 (2.0%) |  |
| **Hypertension or high blood pressure** |  |  |  |  |
| No | 5342 (88.4%) | 1261 (86.0%) | 4081 (89.2%) | <0.001 |
| Yes | 609 (10.1%) | 206 (14.0%) | 403 (8.8%) |  |
| Missing | 93 (1.5%) | 0 (0%) | 93 (2.0%) |  |
| **Overweight or obesity** |  |  |  |  |
| No | 4735 (78.3%) | 1052 (71.7%) | 3683 (80.5%) | <0.001 |
| Yes | 1216 (20.1%) | 415 (28.3%) | 801 (17.5%) |  |
| Missing | 93 (1.5%) | 0 (0%) | 93 (2.0%) |  |
| **Smoking (currently smoking any type of tobacco, including smokeless tobacco)** |  |  |  |  |
| No | 5756 (95.2%) | 1390 (94.8%) | 4366 (95.4%) | <0.001 |
| Yes | 195 (3.2%) | 77 (5.2%) | 118 (2.6%) |  |
| Missing | 93 (1.5%) | 0 (0%) | 93 (2.0%) |  |

^a^ We provided n (column %) for categorical variables and mean (SD) for baseline (acute illness) sickness severity (the only continuous variable ranging from 0 to 10).

^b^ Chi-squared test with Rao & Scott’s second-order correction; Wilcoxon rank-sum test for complex survey samples.

**Supplementary Table 2.** Sociodemographic and Clinical Characteristics of Symptomatic Adults Who Tested Positive (COVID +) vs Negative (COVID -) for SARS-CoV-2 at Enrollment, Unweighted

| **Characteristics^a^** | Total | COVID+ | COVID- | P-value^b^ |
| --- | --- | --- | --- | --- |
|  | (N=1467) | (N=1096) | (N=371) |  |
| **Age (at enrollment)** |  |  |  |  |
| 18 to 34 | 613 (41.8%) | 449 (41.0%) | 164 (44.2%) | 0.973 |
| 35 to 49 | 451 (30.7%) | 343 (31.3%) | 108 (29.1%) |  |
| 50 to 64 | 282 (19.2%) | 212 (19.3%) | 70 (18.9%) |  |
| 65+ | 121 (8.2%) | 92 (8.4%) | 29 (7.8%) |  |
| **Gender** |  |  |  |  |
| Female | 1000 (68.2%) | 722 (65.9%) | 278 (74.9%) | 0.005 |
| Male | 446 (30.4%) | 361 (32.9%) | 85 (22.9%) |  |
| Transgender/Non-binary/Other | 21 (1.4%) | 13 (1.2%) | 8 (2.2%) |  |
| **Ethnicity** |  |  |  |  |
| No, not of Hispanic, Latin or Spanish origin | 1254 (85.5%) | 945 (86.2%) | 309 (83.3%) | 0.320 |
| Yes, of Hispanic, Latin or Spanish origin | 199 (13.6%) | 140 (12.8%) | 59 (15.9%) |  |
| Missing | 14 (1.0%) | 11 (1.0%) | 3 (0.8%) |  |
| **Race** |  |  |  |  |
| White | 1022 (69.7%) | 772 (70.4%) | 250 (67.4%) | 0.961 |
| Black or African American | 110 (7.5%) | 78 (7.1%) | 32 (8.6%) |  |
| Asian | 171 (11.7%) | 126 (11.5%) | 45 (12.1%) |  |
| Other/Multiple | 134 (9.1%) | 98 (8.9%) | 36 (9.7%) |  |
| Missing | 30 (2.0%) | 22 (2.0%) | 8 (2.2%) |  |
| **Educational Attainment** |  |  |  |  |
| Less than high school diploma | 11 (0.7%) | 8 (0.7%) | 3 (0.8%) | 0.816 |
| High school graduate or GED | 93 (6.3%) | 68 (6.2%) | 25 (6.7%) |  |
| Some college but did not complete degree | 216 (14.7%) | 154 (14.1%) | 62 (16.7%) |  |
| 2-year college degree | 123 (8.4%) | 89 (8.1%) | 34 (9.2%) |  |
| 4-year college degree | 490 (33.4%) | 384 (35.0%) | 106 (28.6%) |  |
| More than 4-year college degree | 534 (36.4%) | 393 (35.9%) | 141 (38.0%) |  |
| **Marital Status** |  |  |  |  |
| Never married | 466 (31.8%) | 331 (30.2%) | 135 (36.4%) | 0.021 |
| Married/Living with a part | 840 (57.3%) | 655 (59.8%) | 185 (49.9%) |  |
| Divorced/Widowed/Separated | 161 (11.0%) | 110 (10.0%) | 51 (13.7%) |  |
| **Family Income (pre-pandemic)** |  |  |  |  |
| Less than $10,000 | 93 (6.3%) | 64 (5.8%) | 29 (7.8%) | 0.476 |
| $10,000 to $35,000 | 184 (12.5%) | 129 (11.8%) | 55 (14.8%) |  |
| $35,000 to less than $50,000 | 154 (10.5%) | 109 (9.9%) | 45 (12.1%) |  |
| $50,000 to less than $75,000 | 214 (14.6%) | 160 (14.6%) | 54 (14.6%) |  |
| $75,000 or more | 822 (56.0%) | 634 (57.8%) | 188 (50.7%) |  |
| **Where received COVID test** |  |  |  |  |
| At home testing kit | 84 (5.7%) | 63 (5.7%) | 21 (5.7%) | 0.005 |
| Tent/drive-up testing site | 824 (56.2%) | 654 (59.7%) | 170 (45.8%) |  |
| Clinic including an Urgent Care Clinic | 258 (17.6%) | 176 (16.1%) | 82 (22.1%) |  |
| Hospital | 122 (8.3%) | 87 (7.9%) | 35 (9.4%) |  |
| Emergency department | 70 (4.8%) | 46 (4.2%) | 24 (6.5%) |  |
| Other | 109 (7.4%) | 70 (6.4%) | 39 (10.5%) |  |
| **Health Insurance** |  |  |  |  |
| Private & Public | 57 (3.9%) | 40 (3.6%) | 17 (4.6%) | 0.755 |
| Private only | 1063 (72.5%) | 803 (73.3%) | 260 (70.1%) |  |
| Public only | 300 (20.4%) | 215 (19.6%) | 85 (22.9%) |  |
| None | 47 (3.2%) | 38 (3.5%) | 9 (2.4%) |  |
| **Including yourself, how many adults over the age of 65 are living in your household?** |  |  |  |  |
| None | 1223 (83.4%) | 923 (84.2%) | 300 (80.9%) | 0.54 |
| One | 151 (10.3%) | 104 (9.5%) | 47 (12.7%) |  |
| More than 1 | 93 (6.3%) | 69 (6.3%) | 24 (6.5%) |  |
| **Employed before the pandemic** |  |  |  |  |
| No | 263 (17.9%) | 179 (16.3%) | 84 (22.6%) | 0.024 |
| Yes | 1204 (82.1%) | 917 (83.7%) | 287 (77.4%) |  |
| **Was a non-health essential worker** |  |  |  |  |
| No | 864 (58.9%) | 654 (59.7%) | 210 (56.6%) | 0.831 |
| Yes | 340 (23.2%) | 263 (24.0%) | 77 (20.8%) |  |
| Missing | 263 (17.9%) | 179 (16.3%) | 84 (22.6%) |  |
| **Baseline (acute illness): sickness severity (0-10)** |  |  |  |  |
| Mean (SD) | 5.63 (2.61) | 6.04 (2.36) | 4.43 (2.91) | <0.001 |
| **Asthma (moderate or severe)** |  |  |  |  |
| No | 1267 (86.4%) | 958 (87.4%) | 309 (83.3%) | 0.136 |
| Yes | 200 (13.6%) | 138 (12.6%) | 62 (16.7%) |  |
| **Heart conditions, such as coronary artery disease, heart failure or cardiomyopathies** |  |  |  |  |
| No | 1427 (97.3%) | 1070 (97.6%) | 357 (96.2%) | 0.358 |
| Yes | 40 (2.7%) | 26 (2.4%) | 14 (3.8%) |  |
| **Diabetes** |  |  |  |  |
| No | 1389 (94.7%) | 1045 (95.3%) | 344 (92.7%) | 0.15 |
| Yes | 78 (5.3%) | 51 (4.7%) | 27 (7.3%) |  |
| **Hypertension or high blood pressure** |  |  |  |  |
| No | 1261 (86.0%) | 942 (85.9%) | 319 (86.0%) | 1 |
| Yes | 206 (14.0%) | 154 (14.1%) | 52 (14.0%) |  |
| **Overweight or obesity** |  |  |  |  |
| No | 1052 (71.7%) | 791 (72.2%) | 261 (70.4%) | 0.797 |
| Yes | 415 (28.3%) | 305 (27.8%) | 110 (29.6%) |  |
| **Smoking (currently smoking any type of tobacco, including smokeless tobacco)** |  |  |  |  |
| No | 1390 (94.8%) | 1044 (95.3%) | 346 (93.3%) | 0.33 |
| Yes | 77 (5.2%) | 52 (4.7%) | 25 (6.7%) |  |
| **Subsequent positive COVID-19 test^c^** |  |  |  |  |
| By 3 months | 52 (3.5%) | 32 (2.9%) | 20 (5.4%) | 0.026 |
| By 6 months | 105 (7.2%) | 56 (5.1%) | 49 (13.2%) | <.001 |
| By 9 months | 118 (8.0%) | 69 (6.3%) | 49 (13.2%) | <.001 |
| By 12 months | 172 (11.7%) | 113 (10.3%) | 59 (15.9%) | 0.004 |

^a^ We provided n (column %) for categorical variables and mean (SD) for baseline (acute illness) sickness severity (the only continuous variable ranging from 0 to 10).

^b^ Chi-squared test with Rao & Scott’s second-order correction; Wilcoxon rank-sum test for complex survey samples.

^c^ Subsequent positive COVID-19 test status was identified based on response to two survey questions about whether a new COVID-19 test was done and whether the test results was positive by each time point.

**Supplementary Table 3.** Sociodemographic and Clinical Characteristics of Symptomatic Adults by Latent Transition Analysis Membership, Unweighted

| Characteristics^a^ | Total | Optimal HRQoL | Poor mental HRQoL | Poor physical HRQoL | Poor overall HRQoL | P-value^b^ |
| --- | --- | --- | --- | --- | --- | --- |
|  | (N=1467) | (N=382) | (N=370) | (N=314) | (N=401) |  |
| **Age (at enrollment)** |  |  |  |  |  |  |
| 18 to 34 | 613 (41.8%) | 157 (41.1%) | 203 (54.9%) | 92 (29.3%) | 161 (40.1%) | <0.001 |
| 35 to 49 | 451 (30.7%) | 110 (28.8%) | 116 (31.4%) | 86 (27.4%) | 139 (34.7%) |  |
| 50 to 64 | 282 (19.2%) | 77 (20.2%) | 41 (11.1%) | 86 (27.4%) | 78 (19.5%) |  |
| 65+ | 121 (8.2%) | 38 (9.9%) | 10 (2.7%) | 50 (15.9%) | 23 (5.7%) |  |
| **Gender** |  |  |  |  |  |  |
| Female | 1000 (68.2%) | 204 (53.4%) | 265 (71.6%) | 218 (69.4%) | 313 (78.1%) | <0.001 |
| Male | 446 (30.4%) | 176 (46.1%) | 99 (26.8%) | 93 (29.6%) | 78 (19.5%) |  |
| Transgender/Non-binary/Other | 21 (1.4%) | 2 (0.5%) | 6 (1.6%) | 3 (1.0%) | 10 (2.5%) |  |
| **Ethnicity** |  |  |  |  |  |  |
| No, not of Hispanic, Latin or Spanish origin | 1254 (85.5%) | 331 (86.6%) | 314 (84.9%) | 269 (85.7%) | 340 (84.8%) | 0.849 |
| Yes, of Hispanic, Latin or Spanish origin | 199 (13.6%) | 46 (12.0%) | 55 (14.9%) | 41 (13.1%) | 57 (14.2%) |  |
| Missing | 14 (1.0%) | 5 (1.3%) | 1 (0.3%) | 4 (1.3%) | 4 (1.0%) |  |
| **Race** |  |  |  |  |  |  |
| White | 1022 (69.7%) | 273 (71.5%) | 249 (67.3%) | 229 (72.9%) | 271 (67.6%) | 0.227 |
| Black or African American | 110 (7.5%) | 22 (5.8%) | 24 (6.5%) | 25 (8.0%) | 39 (9.7%) |  |
| Asian | 171 (11.7%) | 52 (13.6%) | 51 (13.8%) | 29 (9.2%) | 39 (9.7%) |  |
| Other/Multiple | 134 (9.1%) | 28 (7.3%) | 33 (8.9%) | 27 (8.6%) | 46 (11.5%) |  |
| Missing | 30 (2.0%) | 7 (1.8%) | 13 (3.5%) | 4 (1.3%) | 6 (1.5%) |  |
| **Educational Attainment** |  |  |  |  |  |  |
| Less than high school diploma | 11 (0.7%) | 2 (0.5%) | 1 (0.3%) | 4 (1.3%) | 4 (1.0%) | 0.001 |
| High school graduate or GED | 93 (6.3%) | 16 (4.2%) | 17 (4.6%) | 28 (8.9%) | 32 (8.0%) |  |
| Some college but did not complete degree | 216 (14.7%) | 42 (11.0%) | 51 (13.8%) | 44 (14.0%) | 79 (19.7%) |  |
| 2-year college degree | 123 (8.4%) | 31 (8.1%) | 21 (5.7%) | 32 (10.2%) | 39 (9.7%) |  |
| 4-year college degree | 490 (33.4%) | 126 (33.0%) | 140 (37.8%) | 92 (29.3%) | 132 (32.9%) |  |
| More than 4-year college degree | 534 (36.4%) | 165 (43.2%) | 140 (37.8%) | 114 (36.3%) | 115 (28.7%) |  |
| **Marital Status** |  |  |  |  |  |  |
| Never married | 466 (31.8%) | 114 (29.8%) | 144 (38.9%) | 71 (22.6%) | 137 (34.2%) | <0.001 |
| Married/Living with a part | 840 (57.3%) | 238 (62.3%) | 192 (51.9%) | 201 (64.0%) | 209 (52.1%) |  |
| Divorced/Widowed/Separated | 161 (11.0%) | 30 (7.9%) | 34 (9.2%) | 42 (13.4%) | 55 (13.7%) |  |
| **Family Income (pre-pandemic)** |  |  |  |  |  |  |
| Less than $10,000 | 93 (6.3%) | 18 (4.7%) | 21 (5.7%) | 15 (4.8%) | 39 (9.7%) | <0.001 |
| $10,000 to $35,000 | 184 (12.5%) | 34 (8.9%) | 40 (10.8%) | 37 (11.8%) | 73 (18.2%) |  |
| $35,000 to less than $50,000 | 154 (10.5%) | 25 (6.5%) | 48 (13.0%) | 30 (9.6%) | 51 (12.7%) |  |
| $50,000 to less than $75,000 | 214 (14.6%) | 43 (11.3%) | 57 (15.4%) | 50 (15.9%) | 64 (16.0%) |  |
| $75,000 or more | 822 (56.0%) | 262 (68.6%) | 204 (55.1%) | 182 (58.0%) | 174 (43.4%) |  |
| **Where received COVID test** |  |  |  |  |  |  |
| At home testing kit | 84 (5.7%) | 24 (6.3%) | 29 (7.8%) | 17 (5.4%) | 14 (3.5%) | 0.001 |
| Tent/drive-up testing site | 824 (56.2%) | 232 (60.7%) | 220 (59.5%) | 156 (49.7%) | 216 (53.9%) |  |
| Clinic including an Urgent Care Clinic | 258 (17.6%) | 52 (13.6%) | 67 (18.1%) | 60 (19.1%) | 79 (19.7%) |  |
| Hospital | 122 (8.3%) | 33 (8.6%) | 20 (5.4%) | 30 (9.6%) | 39 (9.7%) |  |
| Emergency department | 70 (4.8%) | 8 (2.1%) | 9 (2.4%) | 27 (8.6%) | 26 (6.5%) |  |
| Other | 109 (7.4%) | 33 (8.6%) | 25 (6.8%) | 24 (7.6%) | 27 (6.7%) |  |
| **Health Insurance** |  |  |  |  |  |  |
| Private & Public | 57 (3.9%) | 16 (4.2%) | 2 (0.5%) | 24 (7.6%) | 15 (3.7%) | <0.001 |
| Private only | 1063 (72.5%) | 295 (77.2%) | 307 (83.0%) | 210 (66.9%) | 251 (62.6%) |  |
| Public only | 300 (20.4%) | 62 (16.2%) | 55 (14.9%) | 72 (22.9%) | 111 (27.7%) |  |
| None | 47 (3.2%) | 9 (2.4%) | 6 (1.6%) | 8 (2.5%) | 24 (6.0%) |  |
| **Including yourself, how many adults over the age of 65 are living in your household?** |  |  |  |  |  |  |
| No | 1223 (83.4%) | 321 (84.0%) | 335 (90.5%) | 240 (76.4%) | 327 (81.5%) | <0.001 |
| One | 151 (10.3%) | 31 (8.1%) | 24 (6.5%) | 45 (14.3%) | 51 (12.7%) |  |
| More than 1 | 93 (6.3%) | 30 (7.9%) | 11 (3.0%) | 29 (9.2%) | 23 (5.7%) |  |
| **Employed before the pandemic** |  |  |  |  |  |  |
| No | 263 (17.9%) | 62 (16.2%) | 50 (13.5%) | 67 (21.3%) | 84 (20.9%) | 0.031 |
| Yes | 1204 (82.1%) | 320 (83.8%) | 320 (86.5%) | 247 (78.7%) | 317 (79.1%) |  |
| **Was a non-health essential worker** |  |  |  |  |  |  |
| No | 864 (58.9%) | 240 (62.8%) | 247 (66.8%) | 160 (51.0%) | 217 (54.1%) | 0.007 |
| Yes | 340 (23.2%) | 80 (20.9%) | 73 (19.7%) | 87 (27.7%) | 100 (24.9%) |  |
| Missing | 263 (17.9%) | 62 (16.2%) | 50 (13.5%) | 67 (21.3%) | 84 (20.9%) |  |
| **Baseline (acute illness): sick severity (0-10)** |  |  |  |  |  |  |
| Mean (SD) | 5.63 (2.61) | 4.70 (2.38) | 4.91 (2.46) | 6.17 (2.50) | 6.76 (2.50) | <0.001 |
| **Asthma (moderate or severe)** |  |  |  |  |  |  |
| No | 1267 (86.4%) | 343 (89.8%) | 340 (91.9%) | 266 (84.7%) | 318 (79.3%) | <0.001 |
| Yes | 200 (13.6%) | 39 (10.2%) | 30 (8.1%) | 48 (15.3%) | 83 (20.7%) |  |
| **Heart conditions, such as coronary artery disease, heart failure or cardiomyopathies** |  |  |  |  |  |  |
| No | 1427 (97.3%) | 377 (98.7%) | 368 (99.5%) | 296 (94.3%) | 386 (96.3%) | <0.001 |
| Yes | 40 (2.7%) | 5 (1.3%) | 2 (0.5%) | 18 (5.7%) | 15 (3.7%) |  |
| **Diabetes** |  |  |  |  |  |  |
| No | 1389 (94.7%) | 371 (97.1%) | 356 (96.2%) | 289 (92.0%) | 373 (93.0%) | 0.012 |
| Yes | 78 (5.3%) | 11 (2.9%) | 14 (3.8%) | 25 (8.0%) | 28 (7.0%) |  |
| **Hypertension or high blood pressure** |  |  |  |  |  |  |
| No | 1261 (86.0%) | 334 (87.4%) | 340 (91.9%) | 243 (77.4%) | 344 (85.8%) | <0.001 |
| Yes | 206 (14.0%) | 48 (12.6%) | 30 (8.1%) | 71 (22.6%) | 57 (14.2%) |  |
| **Overweight or obesity** |  |  |  |  |  |  |
| No | 1052 (71.7%) | 296 (77.5%) | 279 (75.4%) | 213 (67.8%) | 264 (65.8%) | 0.001 |
| Yes | 415 (28.3%) | 86 (22.5%) | 91 (24.6%) | 101 (32.2%) | 137 (34.2%) |  |
| **Smoking (currently smoking any type of tobacco, including smokeless tobacco)** |  |  |  |  |  |  |
| No | 1390 (94.8%) | 372 (97.4%) | 354 (95.7%) | 300 (95.5%) | 364 (90.8%) | 0.001 |
| Yes | 77 (5.2%) | 10 (2.6%) | 16 (4.3%) | 14 (4.5%) | 37 (9.2%) |  |
| **3 or more of the symptoms at baseline** |  |  |  |  |  |  |
| No | 202 (13.8%) | 73 (19.1%) | 58 (15.7%) | 34 (10.8%) | 37 (9.2%) | 0.001 |
| Yes | 1251 (85.3%) | 305 (79.8%) | 309 (83.5%) | 276 (87.9%) | 361 (90.0%) |  |
| Missing | 14 (1.0%) | 4 (1.0%) | 3 (0.8%) | 4 (1.3%) | 3 (0.7%) |  |
| **3 or more of the symptoms at 3 months** |  |  |  |  |  |  |
| No | 1144 (78.0%) | 342 (89.5%) | 311 (84.1%) | 256 (81.5%) | 235 (58.6%) | <0.001 |
| Yes | 317 (21.6%) | 39 (10.2%) | 57 (15.4%) | 57 (18.2%) | 164 (40.9%) |  |
| Missing | 6 (0.4%) | 1 (0.3%) | 2 (0.5%) | 1 (0.3%) | 2 (0.5%) |  |
| **3 or more of the symptoms at 6 months** |  |  |  |  |  |  |
| No | 1175 (80.1%) | 348 (91.1%) | 329 (88.9%) | 247 (78.7%) | 251 (62.6%) | <0.001 |
| Yes | 284 (19.4%) | 33 (8.6%) | 39 (10.5%) | 64 (20.4%) | 148 (36.9%) |  |
| Missing | 8 (0.5%) | 1 (0.3%) | 2 (0.5%) | 3 (1.0%) | 2 (0.5%) |  |
| **3 or more of the symptoms at 9 months** |  |  |  |  |  |  |
| No | 1199 (81.7%) | 354 (92.7%) | 329 (88.9%) | 254 (80.9%) | 262 (65.3%) | <0.001 |
| Yes | 264 (18.0%) | 26 (6.8%) | 41 (11.1%) | 59 (18.8%) | 138 (34.4%) |  |
| Missing | 4 (0.3%) | 2 (0.5%) | 0 (0%) | 1 (0.3%) | 1 (0.2%) |  |
| **3 or more of the symptoms at 12 months** |  |  |  |  |  |  |
| No | 1193 (81.3%) | 355 (92.9%) | 322 (87.0%) | 257 (81.8%) | 259 (64.6%) | <0.001 |
| Yes | 267 (18.2%) | 27 (7.1%) | 45 (12.2%) | 56 (17.8%) | 139 (34.7%) |  |
| Missing | 7 (0.5%) | 0 (0%) | 3 (0.8%) | 1 (0.3%) | 3 (0.7%) |  |
| **Total number of fatigue symptoms at baseline** |  |  |  |  |  |  |
| Mean (SD) | 2.45 (2.17) | 1.18 (1.43) | 2.04 (1.79) | 2.55 (2.07) | 3.94 (2.25) | <0.001 |
| Median [Min, Max] | 2.00 [0, 8.00] | 1.00 [0, 7.00] | 2.00 [0, 8.00] | 2.00 [0, 8.00] | 4.00 [0, 8.00] |  |
| **Total number of fatigue symptoms at 3 months** |  |  |  |  |  |  |
| Mean (SD) | 1.53 (1.92) | 0.448 (0.894) | 1.21 (1.47) | 1.43 (1.72) | 2.93 (2.30) | <0.001 |
| Median [Min, Max] | 1.00 [0, 8.00] | 0 [0, 5.00] | 1.00 [0, 6.00] | 1.00 [0, 8.00] | 3.00 [0, 8.00] |  |
| **Total number of fatigue symptoms at 6 months** |  |  |  |  |  |  |
| Mean (SD) | 1.64 (1.99) | 0.463 (0.935) | 1.50 (1.72) | 1.67 (1.93) | 2.87 (2.28) | <0.001 |
| Median [Min, Max] | 1.00 [0, 8.00] | 0 [0, 6.00] | 1.00 [0, 7.00] | 1.00 [0, 8.00] | 3.00 [0, 8.00] |  |
| Missing | 1 (0.1%) | 0 (0%) | 0 (0%) | 0 (0%) | 1 (0.2%) |  |
| **Total number of fatigue symptoms at 9 months** |  |  |  |  |  |  |
| Mean (SD) | 1.59 (2.00) | 0.535 (1.06) | 1.40 (1.64) | 1.63 (1.90) | 2.73 (2.42) | <0.001 |
| Median [Min, Max] | 1.00 [0, 8.00] | 0 [0, 7.00] | 1.00 [0, 8.00] | 1.00 [0, 8.00] | 2.00 [0, 8.00] |  |
| Missing | 2 (0.1%) | 1 (0.3%) | 1 (0.3%) | 0 (0%) | 0 (0%) |  |
| **Total number of fatigue symptoms at 12 months** |  |  |  |  |  |  |
| Mean (SD) | 1.63 (2.01) | 0.635 (1.24) | 1.38 (1.66) | 1.56 (1.83) | 2.86 (2.38) | <0.001 |
| Median [Min, Max] | 1.00 [0, 8.00] | 0 [0, 8.00] | 1.00 [0, 8.00] | 1.00 [0, 8.00] | 3.00 [0, 8.00] |  |
| Missing | 3 (0.2%) | 1 (0.3%) | 1 (0.3%) | 1 (0.3%) | 0 (0%) |  |
| **Do you think you had or currently have Long COVID?^c^** |  |  |  |  |  |  |
| No | 770 (52.5%) | 260 (68.1%) | 213 (57.6%) | 161 (51.3%) | 136 (33.9%) | <0.001 |
| Yes | 349 (23.8%) | 37 (9.7%) | 66 (17.8%) | 76 (24.2%) | 170 (42.4%) |  |
| Missing | 348 (23.7%) | 85 (22.3%) | 91 (24.6%) | 77 (24.5%) | 95 (23.7%) |  |
| **2 or more of the symptoms at 3 months** |  |  |  |  |  |  |
| No | 1025 (69.9%) | 322 (84.3%) | 284 (76.8%) | 225 (71.7%) | 194 (48.4%) | <0.001 |
| Yes | 436 (29.7%) | 59 (15.4%) | 84 (22.7%) | 88 (28.0%) | 205 (51.1%) |  |
| Missing | 6 (0.4%) | 1 (0.3%) | 2 (0.5%) | 1 (0.3%) | 2 (0.5%) |  |
| **2 or more of the symptoms at 6 months** |  |  |  |  |  |  |
| No | 1056 (72.0%) | 328 (85.9%) | 302 (81.6%) | 214 (68.2%) | 212 (52.9%) | <0.001 |
| Yes | 403 (27.5%) | 53 (13.9%) | 66 (17.8%) | 97 (30.9%) | 187 (46.6%) |  |
| Missing | 8 (0.5%) | 1 (0.3%) | 2 (0.5%) | 3 (1.0%) | 2 (0.5%) |  |
| **2 or more of the symptoms at 9 months** |  |  |  |  |  |  |
| No | 1072 (73.1%) | 328 (85.9%) | 301 (81.4%) | 213 (67.8%) | 230 (57.4%) | <0.001 |
| Yes | 391 (26.7%) | 52 (13.6%) | 69 (18.6%) | 100 (31.8%) | 170 (42.4%) |  |
| Missing | 4 (0.3%) | 2 (0.5%) | 0 (0%) | 1 (0.3%) | 1 (0.2%) |  |
| **2 or more of the symptoms at 12 months** |  |  |  |  |  |  |
| No | 1057 (72.1%) | 337 (88.2%) | 293 (79.2%) | 220 (70.1%) | 207 (51.6%) | <0.001 |
| Yes | 403 (27.5%) | 45 (11.8%) | 74 (20.0%) | 93 (29.6%) | 191 (47.6%) |  |
| Missing | 7 (0.5%) | 0 (0%) | 3 (0.8%) | 1 (0.3%) | 3 (0.7%) |  |

^a^ We provided n (column %) for categorical variables, mean (SD) for baseline (acute illness) sickness severity (a continuous variable ranging from 0 to 10), and distribution statistics (mean, SD, median, and range) for the number of fatigue symptoms at each time point.

^b^ Chi-squared test with Rao & Scott’s second-order correction; Wilcoxon rank-sum test for complex survey samples.

^c^ This question was asked in the final survey, which was completed ~16 months after the index COVID test date.

**Supplementary Figure 1.** Covariate Balance before and after Application of Inverse Probability Weighting


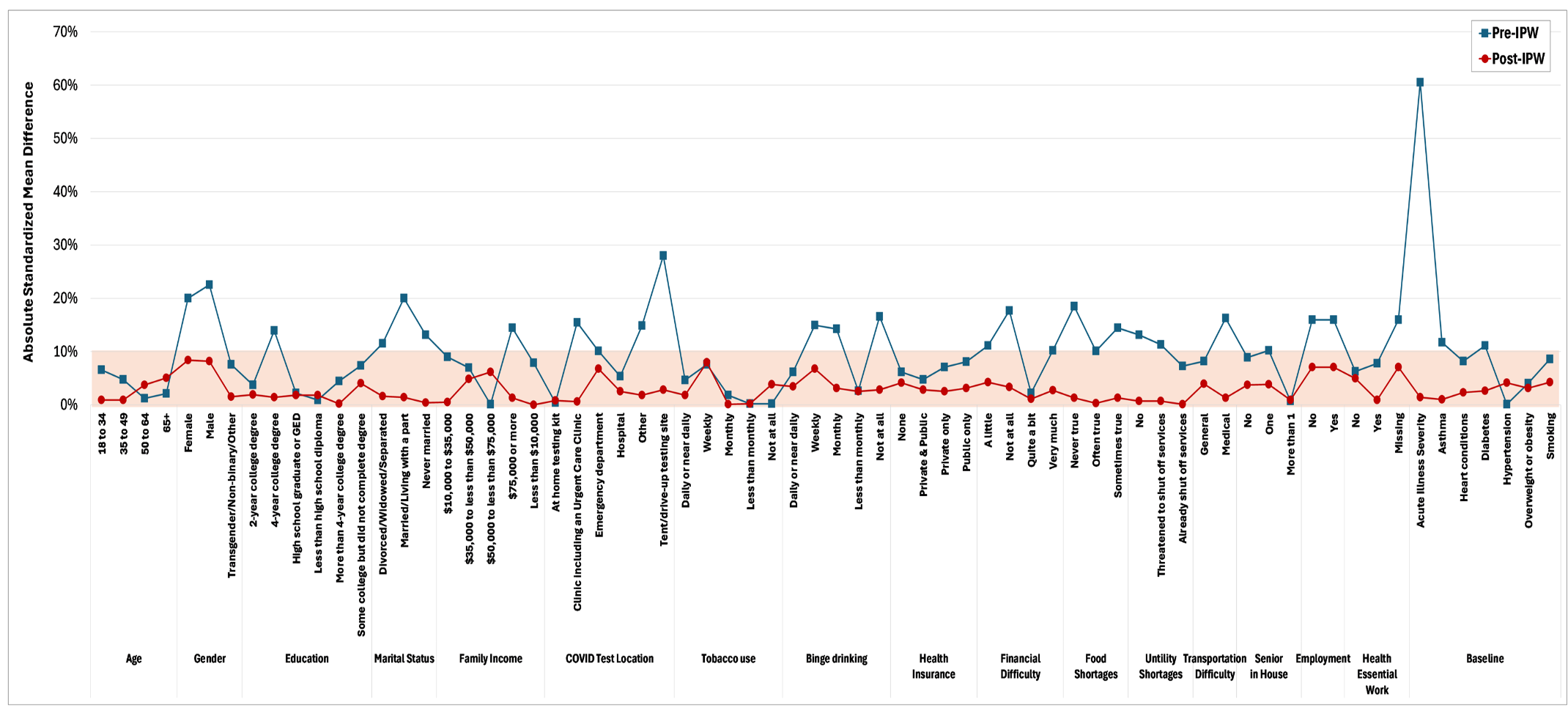


**Supplementary Figure 1 legend:** IPW – Inverse Propensity-score Weighting.

**Alt text:** A graph that depicts absolute standardized mean difference in characteristics between COVID groups (+ and -) before and after application of inverse propensity weighting.

**Supplementary Figure 2.** PROMIS Domain Scores at Baseline, by Latent Class Membership, among All Participants


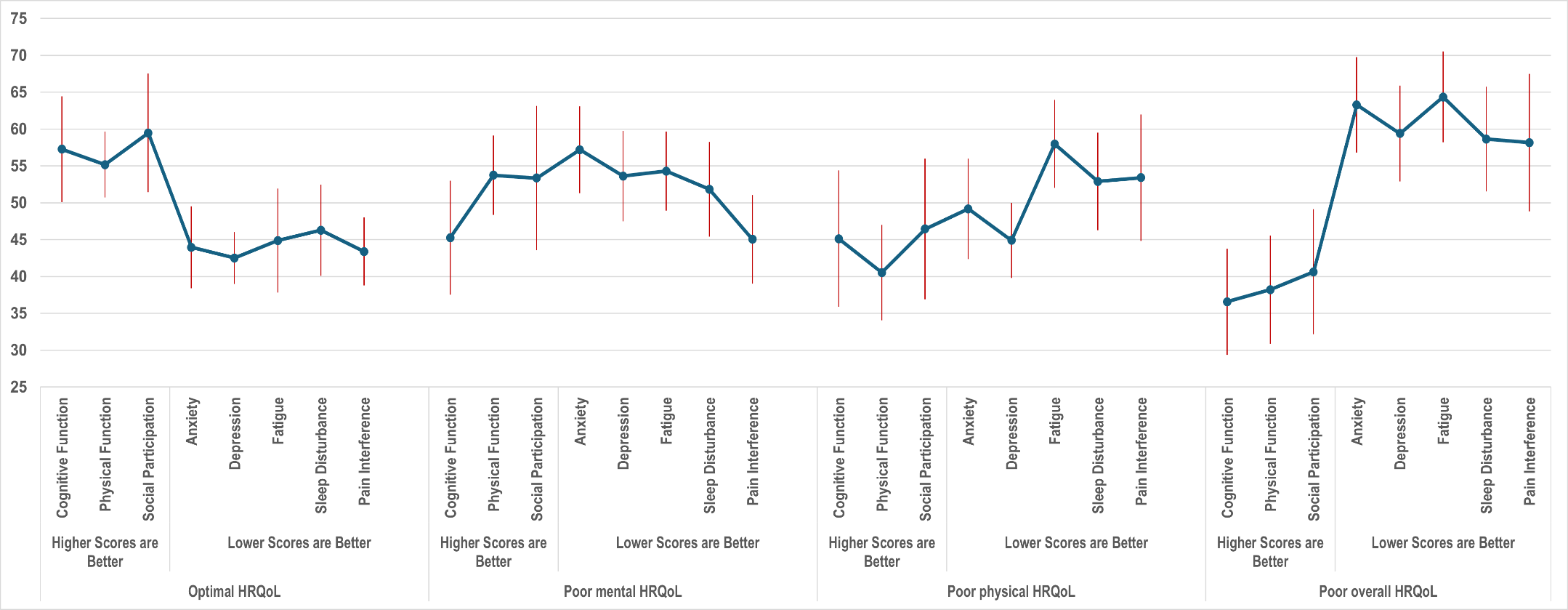


**Supplementary Figure 2 legend:** As latent transition analysis (LTA) was performed irrespective of COVID status (i.e., among all participants), results are shown inclusive of both the COVID+ and COVID- groups. Figure shows unweighted and unadjusted mean PROMIS scores (at baseline, ±standard deviations) on each domain included in the LTA, stratified by final class membership.

**Alt text:** A graph that depicts unweighted and unadjusted mean PROMIS scores (at baseline) on each domain (i.e., cognitive function, physical function, social participation, anxiety, depression, fatigue, sleep disturbance, and pain interference) included in the latent transition analysis stratified by final class membership (i.e., optimal, poor mental, poor physical, and poor overall).

**Supplementary Table 4.** The estimated first order transition probability for latent classes at each time point, to the optimal HRQoL class in the next time point, by COVID-19 status

| **Transition (time *i* to *j*)** | **LTA Class at t_~~i~~_** | **First Order Transition Probability^a^ (*p*_ij_)** | | **Difference in *p*_ij_** |
| --- | --- | --- | --- | --- |
|  |  | **COVID+** | **COVID-** |  |
| Baseline to 3-month | Optimal **HRQoL** | 0.912 | 0.843 | 0.068 |
|  | Poor mental **HRQoL** | 0.271 | 0.148 | 0.122 |
|  | Poor physical **HRQoL** | 0.508 | 0.362 | 0.146 |
|  | Poor overall **HRQoL** | 0.052 | 0.046 | 0.006 |
| 3-month to 6-month | Optimal **HRQoL** | 0.883 | 0.851 | 0.032 |
|  | Poor mental **HRQoL** | 0.121 | 0.088 | 0.033 |
|  | Poor physical **HRQoL** | 0.149 | 0.097 | 0.053 |
|  | Poor overall **HRQoL** | 0.014 | 0.022 | -0.008 |
| 6-month to 9-month | Optimal **HRQoL** | 0.876 | 0.832 | 0.044 |
|  | Poor mental **HRQoL** | 0.206 | 0.136 | 0.070 |
|  | Poor physical **HRQoL** | 0.082 | 0.045 | 0.037 |
|  | Poor overall **HRQoL** | 0.035 | 0.041 | -0.006 |
| 9-month to 12-month | Optimal **HRQoL** | 0.901 | 0.881 | 0.019 |
|  | Poor mental **HRQoL** | 0.093 | 0.082 | 0.011 |
|  | Poor physical **HRQoL** | 0.231 | 0.119 | 0.112 |
|  | Poor overall **HRQoL** | 0.018 | 0.029 | -0.012 |

^a^ The first order transition probability from the LTA classes at t_i_ to optimal **HRQoL** at t_j_. This includes the probability of remaining in the optimal class between time points.

All transition probabilities are based on the GEE model accounting for the latent classes at prior time point and the status of new positive SARS-CoV-2 test results reported by each time point.

**Supplementary Acknowledgements**

**Rush University, Administrative Core & Enrolling Site**

**Study-wide Co- Principal Investigators:** Robert A. Weinstein, MD, Principal Investigator; Michael Gottlieb, MD, Principal Investigator

**Core research team:** Michelle Santangelo, MS, Research Manager; Katherine Koo, MS-HSM, Program Manager; Antonia Derden, BA, Administrative Assistant

**Site Investigators:** Michael Gottlieb, MD, Site Principal Investigator

**Site research team:** Kristyn Gatling, MA, Research Coordinator. Research Assistants: Zohaib Ahmed, MS; Chloe Gomez; Diego Guzman, BS; Minna Hassaballa, BA; Ryan Jerger; Amro (Marshall) Kaadan, ScM

**Yale University, Analytic Core & Enrolling Site**

**Core Investigators:** Principal Investigators: Arjun K. Venkatesh, MD, MBA, MHS; Erica S. Spatz, MD, MHS

**Core research team:** Research Managers: Jeremiah Kinsman, MPH, NREMT, Caitlin Malicki**,** MPH. Statisticians: Zhenqiu Lin, PhD; Shu-Xia Li, PhD; Huihui Yu, PhD; Peizheng Chen, MS; Ji Chen, PhD; Imtiaz Ebna Mannan, MS; Zimo Yang, MS; Mengni Liu, MS

**Site Investigators:** Site Principal Investigators: Arjun K. Venkatesh, MD, MBA, MHS; Erica S. Spatz, MD, MHS. Site Co-Investigator: Andrew Ulrich, MD

**Site Research team:** Research Managers: Jeremiah Kinsman, MPH, NREMT, Caitlin Malicki, MPH. Research Coordinator: Jocelyn Dorney, MPH. Research Assistants: Senyte Pierce, BA; Xavier Puente, BA; Wafa Salah, BA

**University of Washington, Clinical Core & Enrolling Site**

**Core Investigators:** Graham Nichol, MD, MPH, Principal Investigator; Kari A. Stephens, PhD, Co-Principal Investigator

**Core research team:** Jill Anderson, BSN, RN, Clinical Core Program Manager; Mary Schiffgens, MBA, Grant & Finance Manager; Dana Morse, RN, BSN, Research Coordinator; Karen Adams, BA, Regulatory Specialist; Tracy Stober, BA, MA, Patient Representative; Zenoura Maat, Research Assistant

**Site Investigators:** Kelli N. O’Laughlin, MD, MPH, Site Principal Investigator; Nicole L. Gentile, MD, PhD, Co-Investigator

**Site research team:** Research Coordinators: Rachel E. Geyer, MPH; Michael Willis, BSHS; Zihan Zhang, MS, Analyst; Gary Chang, PhD, Senior Biostatistician. Victoria Lyon, MPH, Project Manager. Research Assistants: Robin E. Klabbers, MSc in Medicine, MSc in Global Health; Luis Ruiz, BA; Kerry Malone, BA; Jasmine Park, BSN, RN

**Thomas Jefferson University, Enrolling Site**

**Site Investigators:** Kristin L. Rising, MD, MSHP, Site Principal Investigator; Efrat Kean, MD, Co-Investigator; Anna Marie Chang, MD, MSCE, Co-Investigator

**Site research team:** Nurse Coordinator: Nicole Renzi, RN. Program Manager: Phillip Watts, BA, MM, CCRP. Research Coordinators: Morgan Kelly, BS; Kevin Schaeffer, BS; Dylan Grau, BS; David Cheng, BS; Carly Shutty, BSN; Alex Charlton, BS; Lindsey Shughart, BS; Hailey Shughart, BA, CCRP; Grace Amadio, MD, CCRP; Jessica Miao, BA. Research Assistants: Paavali Hannikainen, BS

**University of California, Los Angeles, Enrolling Site**

**Site Investigators:** Joann G. Elmore, MD, MPH, Site Principal Investigator, Lauren E. Wisk, PhD, Co-Investigator

**Site research team:** Michelle L’Hommedieu, PhD, Site Program Director; Chris Chandler, BA, Research Assistant; Megan Eguchi, MPH, Data Analyst; Kate Diaz Roldan, MPH, Research Assistant; Raul Moreno, BA, Administrative Analyst

**University of California, San Francisco, Enrolling Site**

**Site Investigators:** Robert M. Rodriguez, MD, Site Principal Investigator; Ralph C. Wang, MD, MAS, Site Principal Investigator; Juan Carlos C. Montoy, MD, PhD, Site Principal Investigator

**Site research team:** Robin Kemball, MPH, Program Manager; Research Coordinators: Virginia Chan, MPH; Cecilia Lara Chavez; Angela Wong, BA; Mireya Arreguin, BS

**University of Texas Health Science Center at Houston, Enrolling Site**

**Site Investigators:** Mandy J. Hill, DrPH, MPH, Site Principal Investigator; Ryan Huebinger, MD, Site Principal Investigator.

**Site research team**: Arun Kane, BA, Research Coordinator; Peter Nikonowicz, BA, Research Coordinator; Sarah Sapp, MPH, Research Coordinator

**University of Texas Southwestern Medical Center, Enrolling Site**

**Site Investigators:** Ahamed H. Idris, MD, Site Principal Investigator; Samuel A. McDonald, MD, MS, Co-Investigator

**Site research team:** David Gallegos, BS, Research Coordinator; Katherine Riley Martin, BS, MS, Research Assistant

**Centers for Disease Control and Prevention (CDC)**

**Investigators:** Sharon Saydah, PhD; Ian D. Plumb, MBBS, MSc; Aron J. Hall, DVM, MSPH; Melissa Briggs-Hagen, MD, MPH

**Public Health Seattle King County:** We would like to thank Public Health Seattle King County for their assistance with participant recruitment for this study.

**California Department of Public Health**: We would like to thank the California Department of Public Health for their assistance with participant recruitment for this study.

**CTSI COVID Clinical Research Steering Committee and the CTSI Office of Clinical Research Patient Navigation Team and Bioinformatics Program**: We would like to thank the CTSI COVID Clinical Research Steering Committee and the CTSI Office of Clinical Research Patient Navigation Team and Bioinformatics Program for assistance with study recruitment.

**University of Washington Institute of Translational Health Sciences (ITHS):** We would like to thank the ITHS for support of the REDCap instance and for biomedical informatics resources used by the UW Clinical Core and Enrolling Site to enable study recruitment, which is funded by the National Center for Advancing Translational Sciences of the National Institutes of Health under award number UL1TR002319.
