# Supplementary figures and images for: Association of SARS-CoV-2 With Health-related Quality of Life 1 Year After Illness Using Latent Transition Analysis
Source: Open Forum Infect Dis. 2025 Jun 10;12(6):ofaf278. doi: 10.1093/ofid/ofaf278 (PMC12150399; doi:10.1093/ofid/ofaf278)

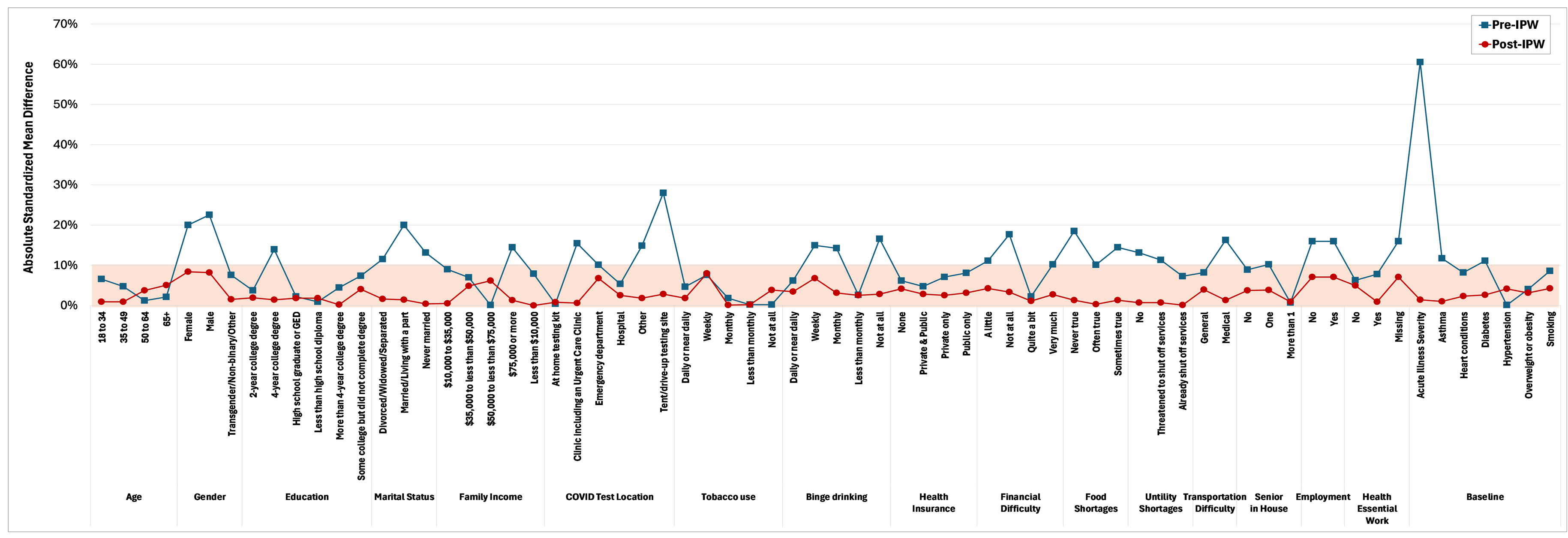

Supplement: ofaf278_Supplementary_Data [file ofaf278_supplementary_data.zip › Supplementary Figure1.tif]

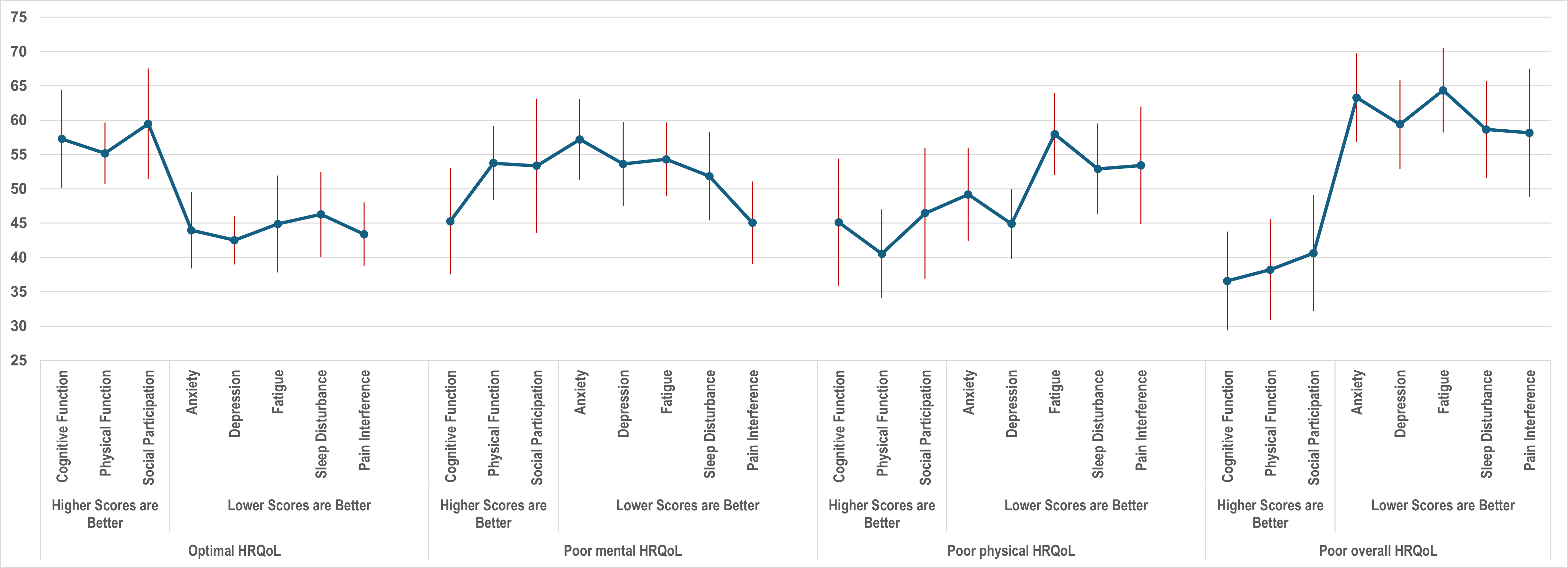

Supplement: ofaf278_Supplementary_Data [file ofaf278_supplementary_data.zip › Supplementary Figure2.tif]
